# Systemic effects of gut microbiota and its relationship with disease and modulation

**DOI:** 10.1186/s12865-015-0083-2

**Published:** 2015-03-26

**Authors:** Jolie TK Ho, Godfrey CF Chan, James CB Li

**Affiliations:** Department of Paediatrics and Adolescent Medicine LKS Faculty of Medicine, The University of Hong Kong, Room L2-01, Laboratory Block, 21 Sassoon Road, Hong Kong, China

## Abstract

The gut microbiota makes up the majority of the human bacterial population, and although the gut microbiota resides in the intestines, it is able to exert systemic effects. Therefore, many diseases and conditions could be impacted by the gut microbiota when its composition is imbalanced, otherwise known as dysbiosis. However, apart from understanding the illnesses, we must also try to understand the intestinal flora itself to move forward and develop potential treatments.

## Review

### Introduction

Although the word “bacteria” is frequently associated with negative connotations of infection and disease, there is in fact an abundance of bacteria that is beneficial for the human body. These certain bacteria are microbiota, which have a commensal relationship with the body—the body gives the bacteria a place to flourish, and in return, the bacteria offer protection and help with regulation. The entire human microbiota has a total of 10^14^ bacterial cells, which is 10 times the number of human cells in the body [1]. Some examples of the locations of microbiota include the skin, the vagina, the oral cavity, but most prominently, the intestines, where gut microbiota reside.

Gut microbiota comprises approximately 70% of the entire microbiota population, and is dominated by the *Bacteroidetes* and *Firmicutes* phyla. Other phyla that exist in gut microbiota in smaller quantities include *Proteobacteria, Verrucomicrobia, Actinobacteria, Fusobacteria,* and *Cyanobacteria* [2]*.* Gut microbiota aids in food digestion and also helps with the production of some vitamins like vitamins B and K, which are essential towards cell metabolism and blood coagulation by modifying proteins to allow binding to calcium ions. Furthermore, gut microbiota can combat harmful microorganisms by creating a barrier effect in the immune system. The importance of acquiring microbiota has been emphasized in studies with germ-free animals, where it was found that commensal organisms are required for the development of a fully functional immune system [3]. Babies delivered by Cesarean section are at higher risk for immune-mediated diseases because they did not undergo initial microbial colonization from the vaginal canal [4]. The microbiota not only plays a role in the local intestinal immune system, but also in systemic immune responses [5].

Changes in microbiota diversity and balance can lead to physiological changes that are not restricted to the gastrointestinal system. One of the modes by which gut microbiota impacts other parts of the body is controlled by intestinal permeability. Pathogen overgrowth and certain models of stress promote the loss of the intestinal barrier, thus increasing intestinal permeability, allowing for gut microbiota to travel across the intestinal epithelium and into systemic circulation. This phenomenon is often referred to as “leaky gut” syndrome, and it enables gut microbiota to impact the entire body and immune system [6]. Therefore, a healthy balance of gut microbiota is crucial not only for proper digestive functioning, but also for a strong immune system. It follows that imbalances and dysregulation of gut microbiota can lead to a host of different diseases. Some different types include autoimmune, hyper-immune, cardiovascular, chronic, neurological, cancerous, psychiatric diseases, and many more.

This review will cover some of the diseases related to microbiotal dysbiosis, as well as highlight ways that can be used to further expand our current knowledge. Furthermore, this review will consider the modification of gut microbiota in the body to help counter microbial imbalance, and potentially act as a form of treatment.

## Diseases

### Autoimmune

An example of an autoimmune disease influenced by gut microbiota is Type 1 diabetes (T_1_DM), or juvenile diabetes. Studies comparing germ-free and gnotobiotic (populated with specific microbes) mice have revealed that T_1_DM is among the diseases affected by reduced numbers of commensal bacteria [7], especially low numbers of butyrate-producing bacteria such as those from the *Firmicutes* phylum, leading to an altered ratio between *Bacteroidetes* and *Firmicutes* bacteria [8]. The imbalance between these two dominant phyla could lead to more physiological problems for the patients. A study has also shown that diabetic patients younger than 2.9 years have less bacteria from *Clostridial* clusters IV and XIVa, which also produce butyrate, hence corroborating data from the mice studies [6].

Inflammatory bowel disease (IBD) is a gastrointestinal disorder also due to autoimmune dysregulation. IBD is a spectrum of chronic diseases marked by recurring inflammation of the intestinal mucosal lining. Two main phenotypes of IBD are Crohn’s disease (CD) and ulcerative colitis (UC), and both have been shown to be linked to gut microbiota dysbiosis. Various studies claim that IBD exhibits significant decrease in microbial diversity, increased bacterial count, and increase in detrimental bacteria [9]. Studies indicate that UC is characterized by a decline in *Firmicutes* and *Bacteroidetes*, like in T_1_DM, and an unusual increase in *Proteobacteria*. Also like T_1_DM, UC has also been associated with a loss of bacteria from butyrate-producing *Clostridial* cluster XIVa [10]. In CD, the disease was mainly observed in areas containing the highest concentrations of bacteria [11]. Furthermore, a metabonomic study by Bjerrum et al. has shown that while UC is marked by a decrease in *Clostridial coccoides* of *Clostridial* cluster XIVa, CD showed a decrease in *Faecalibacterium prausnitzii.* Interestingly, both *C. coccoides* and *F. prausnitzii* are important in the formation of short chain fatty acids, which includes butyrate. Although decreased butyrate stems from these specific bacterial deficiencies, decreased butyrate in itself can perpetuate the cycle of chronic inflammation and microbiotal dysbiosis in UC and CD. Therefore, these two phenotypes of IBD ultimately both end in dysbiosis even with decrease- in different species of bacteria.

There has also been interest shown in the link between genetics, microbiota, and IBD. In one study, the microbiota of siblings of CD patients were studied and compared to the patients’ microbiota. It was shown that siblings of CD patients have a higher risk of developing CD and, like the CD patients, show signs of fecal dysbiosis [12]. Furthermore, since CD is caused by interactions between genetic and environmental factors, gut microbiota plays a role in the disease. The study confirmed microbiota alterations in CD patients, for example reduction in diversity, decrease in *Ruminococcaceae*, and increase in *Enterbacteriaceae* [13].

Dysbiosis is also related to the development of CD and UC in children, which becomes readily apparent when looking at the methods used to treat pediatric IBD. One commonly used treatment for pediatric CD is exclusive enteral nutrition (EEN)—the total replacement of normal diet with liquid diet/ formula during the duration of treatment. Seeing as gut flora can be affected by environmental factors such as diet, the effectiveness of EEN suggests a relationship between microbiotal dysbiosis and the development of CD.

Other autoimmune conditions such as allergies have also been shown to be influenced by gut microbiota. Low microbial diversity has been observed to precede allergic diseases [14]. A possible explanation for the low microbial diversity is linked to the hygiene hypothesis of allergy. In the context of microbiota, the hypothesis suggests that excessively hygienic practices impede the development of a diverse and balanced gut microflora in infants, resulting in irregular immune development and hence the emergence of allergic disease.

Two longitudinal studies by Azad et al. point towards a relationship between gut microbiota and the hygiene hypothesis. The first study looked at the influence of pets and siblings on microbiota composition and diversity and found that microbiota richness and diversity was increased in infants living with pets, but decreased in those living with older siblings, particular in relation to levels of *Bifidobacteriaceae* and *Peptostreptococcaceae*. The second study investigated food sensitization and gut microbiota, and found that low gut microbiota richness paired with an increased ratio between *Enterobacteriaceae* and *Bacteroidaceae* are linked to food sensitization. Thus, gut microflora composition in infants coupled with the hygiene hypothesis seems to be a reasonable connection.

### Psychiatric

There is known to be bidirectional communication between the gut and the brain in the gut-brain axis. Established pathways of communication between the gut and the brain include the autonomic nervous system (ANS) and the enteric nervous system (ENS) [15]. In addition, there has been increasing interest in the microbiota-gut-brain axis ever since the observation that oral antibiotics and laxatives improved cases of hepatic encephalopathy [16]. The microbiota-gut-brain axis is also a point of interest for its role in both inducing and treating psychiatric stress-related conditions such as depression and anxiety.

Stress is chiefly monitored by the hypothalamic-pituitary-adrenal (HPA) axis. Depression and anxiety have both been linked to unregulated HPA axes and over-secretion of corticotropin-releasing factor (CRF), and in turn, adrenocorticotropic hormone (ACTH) in the presence of stress [17]. This relates to gut microbiota because stress is known to increase intestinal permeability, allowing bacteria to travel across the intestinal mucosa and interact with the nervous system. In fact, a 2004 report established a direct link between microbiota and the HPA axis [15], connecting microbiota with depression and anxiety. This link was further supported more recently in April 2014 in a study involving germ-free (GF) and specific pathogen free (SPF) rats [18]. It was found that in social experiments, GF rats spent less time sniffing unknown partners, indicating higher levels of stress in unfamiliar social situations. Furthermore, GF rats had higher CRF mRNA expression in the hypothalamus and lower dopaminergic turnover rates in the frontal cortex, hippocampus, and striatum. However, the GF rats did not have any sensorimotor differences from the SPF rats [18], which isolate the impact of gut microbiota chiefly to the HPA axis. This evidence supports that an absence, and possibly imbalance, of gut microbiota impacts behavioral responses to acute stress, contributing to depression and anxiety.

Besides the connection between gut microbiota and the brain via the HPA axis, there has been evidence attributing microbiota-gut-brain communication to the vagus cranial nerve [19,20]. A study involving mice proved that chronic treatment with *lactobacillus rhamnosus* altered GABA mRNA in the brain and reduced stress-induced corticosteroid, but that these changes were not observed in vagotomized mice [21]. However, further investigation should be carried out with regards to this specific pathway to obtain more definitive knowledge.

In terms of pediatrics, one of the more frequently studied psychiatric conditions in relation to gut microbiota has been autism. It has been noted that autism—a developmental disorder marked by impaired social interactions and restricted/repetitive behavior—tends to present with digestive issues. Finegold et al. found that autistic children have higher counts of *Clostridial* bacteria than control children, including nine species of *Clostridium* not found in the controls. In addition, it was found that autistic children have increased *Bacteroidetes,* and decreased *Firmicutes* and *Bifidobacterium* species. Although correlation does not necessitate maen causative association, such findings provide new insight towards the studying of autism.

### Cancers

Cancer has a variety of causes, such as genetics, UV exposure, radiation exposure, carcinogens, and diet and physical activity. It has also been found that gut microbiota may be related to the development of some cancers, such as colorectal cancer (CRC). CRC is cancer of the colon, rectum, and anus in the form of malignant tumors. Although the development of CRC is influenced by genetic factors such as damaged DNA and genetic instability, environmental factors that impact the gut microbiota may also promote CRC development [22]. This has been supported by mouse models, where fecal microbiota from CRC patients and healthy individuals were transplanted into GF mice and induced different levels of tumorigenesis in the mice. With regards to specific bacterial types involved in the tumorigenesis, gram-negative bacteria had the highest correlation while gram-positive bacteria such as *Clostridial* cluster XIVa were strongly negatively correlated with tumors [23]. Even though the mice were transplanted with distinct microbial populations from different human patients, they all underwent structural changes and the extent of these changes was related to tumor incidence. The study concluded that the initial structure of gut microbiota affects susceptibility to colonic tumorigenesis [23]. Obesity, another prominent risk factor for cancer, has been associated with microbiotal dysbiosis, and could result in physiological changes towards cancer. Microbial metabolism has also been speculated to be related to cancer development [24].

Hepatocellular carcinoma (HCC) is another instance of cancer impacted by gut microbiota. Liver cirrhosis and HCC are not unusual in end-stage chronic liver disease, but the molecular mechanisms relating HCC and liver disease are still not completely clear [25]. However, it was recently discovered that increased translocation of gut microbiota is characteristic of chronic liver disease [26], and that gut microbiota may be the main source of portal vein lipopolysaccharide (LPS), thus promoting tumorigenesis [25]—a theory also supported by the earlier example of a high correlation of gram-negative bacteria in CRC development. It has been speculated that LPS from the gram-negative bacteria promotes hepatocarcinogenesis but does not actually change the gut microbiota composition [26].

However, there exists some controversy over the effect of gut microbiota in the early stages of hepatocarcinogenesis. Yu et al. found a link between gut microbiota and TLR4 to tumor initiation. On the other hand, Dapito et al. concluded that gut microbiota and TLR4 do not have a role in initiating HCC but rather promote it [26]. Dapito et al. also found that even though gut sterilization prevented the development of HCC, it did not lead to regression of already existing tumors. Therefore, although some information is known about gut microbiota relating to cancer, much remains to be clarified, particularly in terms of HCC, before it can be considered conclusive.

## Treatments

Using the information known about gut microbiota imbalances in relation to disease, treatments involving microbiota may be used in attempts to treat these illnesses.

### Biotics

#### Probiotics

Probiotics are dietary supplements that contain live bacteria to add to and strengthen the already existing gut microbiota, a common example being *lactobacilli* in dairy products such as yoghurt.

Probiotics can be used in a wide variety of diseases related to microbiota, including depression and anxiety. As mentioned earlier, a mouse model study investigated the effects of *lactobacillus rhamnosus* on GABA, and showed that chronic treatment with *lactobacillus rhamnosus* caused changes in GABA mRNA in the brain and reduced stress-induced corticosteroid [21]. Other human studies have also reported that altering gut microbiota with probiotics may lead to change in brain function and even in subjective reports of mood [27].

For diabetes, the mechanisms by which anti-diabetic probiotics function may be related to reduction of oxidative stress and inflammation with modification of gut microbiota [28]. Probiotics may also affect the enteric immune system by producing IgA or influencing the release of anti-inflammatory cytokine. So far, the most common probiotics suggested for diabetes are *lactobacillus* and *bifidobacterium* [28], which may improve the absorption of antioxidants for protection against damage by free radicals in the body.

For IBD, probiotics have been shown to be only mildly effective. Probiotics have some effect in treating UC, but no such similar results have been found in treating CD. This could be because CD is a disease made up of many different factors with varying genetics, phenotypes, and severity [Guandalini]. On the other hand, UC does not seem to be as common in family histories. Although CD and UC are both inflammatory bowel diseases, probiotics cannot treat them with equal effectiveness, showing that changing one parameter may not be enough to cure the disease. Diet and other environmental components need to be taken into account. Unless the disease is completely and undoubtedly caused by microbiotal dysbiosis alone, probiotics may be useful but the disease should still be approached holistically.

Although the use of probiotics has been suggested for many types of diseases, the ideal probiotic strain for each type has not yet been identified. Furthermore, concrete data regarding the safety of probiotic usage is still not entirely sufficient [29]. These two areas should be improved upon to make the usage of probiotics a more effective.

### Prebiotics

Unlike probiotics, prebiotics are not live preparations, but instead are food ingredients that may be fermented but not digested. The fermentation of prebiotics can benefit the host by stimulating growth and activity in intestinal microbial species. Prebiotics are not absorbed by the small intestine, and their fermentation allows endogenous bacteria to produce energy and metabolic substrates. So far, the main prebiotics include inulin-type fructans (ITF) and short-chain fructo-oligosaccharides (scFOS) [29]. Different studies involving obese women and gnotobiotic mice have shown that ITF and scFOS stimulate *Bifidobacteria,* which benefit the host by reducing intestinal endotoxin concentration and improving glucose tolerance and inflammation [30,31].

### Synbiotics

Recently, there have been attempts at using pre- and probiotics simultaneously as treatment. The combination of pre- and probiotics is a new approach called synbiotics. A study on elderly fecal microbiota supports the ability of synbiotics to modulate intestinal flora. The effect of two prebiotics and two probiotics, both individually and in synbiotic combinations, were investigated. The synbiotic combinations were shown to increase the *Bifidobacterium* and *Lactobacillus* count in elderly individuals [32]. Although the study did not try to treat a specific condition, it shows that synbiotics could be entertained as a possibility for treatment rather than just pre- and probiotics individually.

In fact, there is currently a clinical trial underway which will try to treat chronic kidney disease (CKD) with synbiotics by targeting uremic toxin synthesis. There have also been a few clinical trials that have tried to alleviate irritable bowel syndrome (IBS) with synbiotics, and these trials have shown some promise [29,33]. Otherwise, there is still limited data on the efficacy and safety of synbiotics for human diseases.

#### Fecal microbiota transplantation (FMT)

Fecal microbiota transplantation (FMT) is another method that can be used to treat diseases due to gut microbiota dysbiosis. Since only 60% of the human microbiota is stable and durable [34], there is room left for microbiota modulation. Up until now, FMT has mainly been used to treat *Clostridium difficile* infection (CDI) with high success. Van Nood et al. emphasized the effectiveness of FMT compared to Vancomycin: FMT was curative for 81% of patients while Vancomycin, which originated from soil bacteria, was only effective for 31% [35,36]. It has been hypothesized that FMT helps with the recovery of a bacteria that can resist the colonization of *C. difficile,* but it is not completely clear exactly how this occurs Another possibility, as shown in a recent study, is that FMT leads to an increase in secondary bile salts, suggesting that bile salt metabolism is important in limiting CDI [37].

Prior to FMT, the majority of the gut microbiota consisted of *Proteobacteria,* but after FMT, the *Proteobacteria* count decreased and there is a higher diversity of *Firmicutes* and *Bacteroidetes* [35,37]. It has also been found that post-FMT, the recipient gut microbiota composition tends towards that of the donor’s with a strong representation of *Firmicutes, Clostridia,* and *Bacilli*.

Besides CDI, a study was conducted on the effect of FMT on patients with chronic active ulcerative colitis (UC). The aim was to see if UC patients could improve with FMT and if the microbiotal dysbiosis in UC could be reversed. Although all the patients experienced short-term improvement within the first two weeks of FMT, none of them achieved complete remission or long-term improvement [34]. Therefore, it was concluded that microbiotal dysbiosis is only a secondary cause in UC, unlike in CDI. Furthermore, a separate study involving mice tried to use FMT to determine whether resistance to food-borne listeriosis depended on the murine gut microbiota. It was found that FMT increased neither susceptibility nor enhancement of listeriosis [30]. These examples highlight the importance of clarifying which diseases are caused primarily by microbiotal imbalances, otherwise FMT may not prove effective.

Although FMT has technically been in practice as a therapeutic method for millennia, it has only been brought to medical attention in recent years. Because gut microbiotal dysbiosis can contribute to obesity, metabolic syndromes, etc., FMT could put a patient at risk for these diseases as complications [38]. Despite FMT’s high success rate in treating CDI, there is still insufficient data for wider usage of FMT. Another challenge facing the use of FMT is that fecal donors must be extensively screened, which could slow down treatment. Some even believe that fecal transplants will soon become outdated after the medical community learns to identify only the necessary specific microorganisms needed to fight different diseases [38]. Even though FMT is gaining in popularity, some safety concerns still remain, and the US Food and Drug Administration (FDA) requires an investigational new drug (IND) application for its use in treating all other gastrointestinal and non-gastrointestinal diseases [39].

Studies comparing the use of FMT in children and adults have shown that children treated with FMT for *C. difficile* have had restoration of bowel function. Again, this could simply be due to the nature of *C. difficile* being primarily caused by microbiotal dysbiosis of a specific bacteria. A study showed some efficacy in treating pediatric UC with FMT; however, with their study being the first of its kind and with only nine cases studied, more data would provide confirmation of this finding.

### Dietary alterations

The gut microbiota is susceptible to modulation by environmental factors [34], such as diet. Development of gut microbiota starts at birth, when the baby is exposed to a complex array of bacteria in the birth canal. A baby’s gut microbiota closely resembles its mother’s, as shown by studies involving mice, and stabilizes at around the age of one [40]. As time goes on, a child’s initial colonization of gut microbiota is influenced by diet and, as a result, varies greatly between individuals. The adaptive nature of gut microbiota is further supported by another study that compared fecal samples of children in Europe and rural Africa. The African children have high-fiber diets due to reliance on agricultural food sources, while the European children have diets high in sugar, starch, and fat but low in fiber. The African children were found to have high numbers of *Bacteroidetes* but lacked *Firmicutes*, but had an abundance of bacteria from the genus *Prevotella* and *Xylanibacter* which the European children lacked completely [41].

Therefore, diet could play a large role in helping to rebalance gut microbiota. Although diet has been shown to impact the gut microbiota, more research could be carried out with regards to what kinds of diets are most beneficial for different pattern of microbial imbalances associated with specific conditions.

## Conclusion and future outlooks

Although gut microbiota reside in the intestines, its systemic effects are significant. It has become evident that microbiotal dysbiosis contributes to many of these systemic effects. However, further investigation is needed to truly clarify whether the relationship between microbiotal dysbiosis and diseases is a causal one. For example, patients with T_1_DM cannot process glucose and must maintain special diets, which could lead to altered microbiota composition being a consequence rather than a factor. It is imperative to distinguish between causal effects, correlations, and consequences when dealing with gut microbiota and disease, and more work is needed in this area.

Also, although there exists general information on the mechanisms and actions of gut microbiota, more in depth investigation is needed to genuinely understand its role in specific cases. So far, although a few attempts at manipulating gut microbiota as therapy have been met with some success, there are conflicting results which makes interpretation difficult in arriving at a consensus [25,26,42].

Interest in gut microbiota has increased exponentially in recent years, with yield of more insights, discoveries, and revelations than ever before. However, more in depth exploration in would help to enhance the understanding of gut microbiota than ever before.

## Abbreviations

(T_1_DM): Type 1 diabetes
(IBD): Inflammatory bowel disease
(CD): Crohn’s disease
(UC): Ulcerative colitis
(GF): Germ-free
(SPF): Specific pathogen free
(CRC): Colorectal cancer
(HCC): Hepatocellular carcinoma
(FMT): Fecal microbiota transplantation
(CDI): *Clostridium difficile* infection
